# Kupe Virus, a New Virus in the Family *Bunyaviridae*, Genus *Nairovirus*, Kenya

**DOI:** 10.3201/eid1502.080851

**Published:** 2009-02

**Authors:** Mary B. Crabtree, Rosemary Sang, Barry R. Miller

**Affiliations:** Centers for Disease Control and Prevention, Fort Collins, Colorado, USA (M.B. Crabtree, B.R. Miller); Kenya Medical Research Institute, Nairobi, Kenya (R. Sang)

**Keywords:** Nairovirus, Kupe virus, Dugbe virus, arbovirus, tick-borne virus, Kenya, research

## Abstract

We have previously described isolation and preliminary identification of a virus related to Dugbe virus (DUGV), family *Bunyaviridae*, genus *Nairovirus*. Six isolates of the virus were obtained from pools of *Amblyomma gemma* and *Rhipicephalus pulchellus* ticks collected from hides of cattle in Nairobi, Kenya, in October 1999. We report results of further characterization of this virus, including growth kinetics in cell culture and full-length genome sequencing and genetic characterization, which show it to be distinct from DUGV. We suggest that this is a new virus in the family *Bunyaviridae*, genus *Nairovirus*, and we propose that it be designated Kupe virus.

## Abstract 2

One-sentence summary for table of contents: A new nairovirus isolated from ticks collected from cattle hides was characterized.

The genus *Nairovirus* in the family *Bunyaviridae* comprises 7 species groups containing primarily tick-borne viruses, some of which have been identified as human or animal pathogens. The genome of the nairoviruses consists of 3 segments of negative-sense, single-stranded RNA, small (S), medium (M), and large (L), which encode the nucleocapsid protein, glycoproteins (Gn and Gc), and viral polymerase, respectively. Additionally, an M segment–encoded nonstructural protein, NS_M_, was recently identified in the nairovirus Crimean-Congo hemorrhagic fever virus (CCHFV) (*1*). In recent years, nucleotide and amino acid sequence information has become available so that additional characterization of these viruses is possible, including further analysis of relationships among members of the genus. Full-length sequence data are now available for CCHFV, Hazara virus (HAZV) and Dugbe virus (DUGV), and partial sequences are available for many other members of the genus. CCHFV, which ranges from sub-Saharan Africa to western People’s Republic of China, is currently the most well characterized member of the genus. DUGV, also well characterized, is commonly isolated in surveillance studies conducted in Africa and appears to be endemic in most of the drier parts of this continent. DUGV is transmitted by ticks to vertebrates, including humans, and causes a mild febrile illness and thrombocytopenia (*2*).

In a recent survey of ticks infesting market livestock in Nairobi, Kenya, we identified 26 isolates of DUGV and additionally obtained several isolates of a virus that was identified as a nairovirus related most closely to DUGV (*3*). We report further characterization of the K611 isolate of this virus, including the full-length genome. Our findings suggest that this is a new virus in the genus *Nairovirus*, and we propose that it be designated Kupe virus (Kupe is the Kiswahili word for tick).

## Materials and Methods

Isolates of viruses were obtained from pools of ticks collected at abattoirs in Nairobi, Kenya, as described (*3*). The K611 isolate used in this study was obtained from a pool of *Amblyomma gemma* ticks in October 1999.

### Characterization of Viruses in Cell Culture and Mice

Growth of Kupe virus and DUGV was tested in Vero (African green monkey kidney), LLC-MK_2_ (rhesus monkey kidney), BHK (baby hamster kidney), SW-13 (human adrenal cortex carcinoma), HeLa (human cervical adenocarcinoma), HUH-7 (human hepatocarcinoma), and C6/36 (*Aedes albopictus* mosquito) cells in culture. Growth kinetics of the 2 viruses were compared in a 13-day growth curve in which cells were infected at a multiplicity of infection of 0.01 and aliquots removed daily. Virus titers were assayed on Vero cell monolayers in 6-well plates by using a published double-overlay method (*4*). Second overlays containing neutral red were added at 6-days postinfection.

### Nucleic Acid Sequencing

Viruses to be sequenced were amplified in Vero cells, and viral RNA was extracted from cell culture supernatant by using the QIAamp Viral RNA Mini Kit (QIAGEN, Valencia, CA, USA). Reverse transcription–PCR was conducted by using the Titan One Tube Reverse Transcription–PCR system (Roche, Indianapolis, IN, USA). Amplified products were purified by agarose gel electrophoresis, and DNA fragments were extracted by using the MinElute Gel Extraction Kit (QIAGEN). Purified DNA fragments were sequenced by using the BigDye 3.1 kit (PE Applied Biosystems, Foster City, CA, USA) and analyzed by using a model 3130 automated sequencer (PE Applied Biosystems). Both strands of the DNA were sequenced.

The full-length genome of Kupe virus isolate K611 was sequenced, beginning with fragments amplified by Nairobi sheep disease virus (NSDV)–specific primers or DUGV-specific primers from each segment. Full-length sequence was obtained by using a previously described method of primer walking and the 5′/3′ Rapid Amplification of cDNA Ends (RACE) Kit (Roche), which was used to determine the sequence of the segment ends (*5*). Fragments of the S (nt 413–916), M (nt 408–2372), and L (nt 6656–8185) segments from other Kupe virus isolates were also sequenced for comparison (*3*). Additionally, fragments of the S, M, and L segments from isolates of DUGV collected in 1999 from the Nairobi abattoirs were sequenced by using primers designed from the published sequence of DUGV (*3*).

### Genome Characterization and Comparison with Other Viruses

The nucleotide sequence of each segment of the Kupe virus genome was analyzed for open reading frames (ORFs) by using the EditSeq module of Lasergene (DNASTAR, Inc., Madison, WI, USA) and translated into deduced amino acid sequence. Identification of protein motifs and potential sites for glycosylation was accomplished by using Prosite (http://ca.expasy.org/prosite), psi-BLAST and CDS-BLAST (www.ncbi.nlm.nih.gov/BLAST), NetOGlyc 3.1, and MOTIFS in the Wisconsin Package version 11.1.2 (*6*,*7*). Nucleotide and amino acid sequences were compared with DUGV, CCHFV, NSDV, and HAZV sequences. GenBank accession numbers for sequences used in this study are listed in Table 1 or in the text below. Sequence alignments were performed by using the PILEUP and GAP programs in the Wisconsin Package. Sequence identities were calculated by using the GAP program (Wisconsin Package) or MegAlign (Lasergene; DNASTAR, Inc.). Phylogenetic analysis of alignments was conducted by using the maximum parsimony method with 500 bootstrap replicates in MEGA, version 3.1 (www.megasoftware.net).

**Table 1:** Virus sequences used in phylogenetic comparisons*

Genome segment	Virus	Strain	GenBank nucleotide accession no.	GenBank amino acid accession no.
Small	Dugbe	ArD44313	AF434161	AAL73396
	Dugbe	KT281/75	AF434165	AAL73400
	Dugbe	IbAr1792	AF434164	AAL73399
	Dugbe	IbH11480	AF434163	AAL73398
	Dugbe	ArD16095	AF434162	AAL73397
	Nairobi sheep disease	RV082	AF504294	AAM33324
	Hazara	JC280	M86624	AAA43842
	Crimean-Congo hemorrhagic fever	IbAr10200	U88410	AAB48501
	Kupe	K611	EU257626	NA
Medium	Dugbe	ArD44313	M94133	AAA42974
	Hazara	JC280	DQ813514	ABH07417
	Crimean-Congo hemorrhagic fever	IbAr10200	AF467768	AAM48106
	Kupe	K611	EU257627	NA
Large	Dugbe	ArD44313	U15018	AAB18834
	Hazara	JC280	DQ076419	AAZ38668
	Crimean-Congo hemorrhagic fever	IbAr10200	AY389508	AAQ90157
	Kupe	K611	EU257628	NA

*NA, not available.

## Results

Viruses were isolated from pools of ticks collected from livestock driven to market at 2 abattoirs in Nairobi, Kenya, as described (*3*). Several isolates made from pools of *A*. *gemma* and *Rhipicephalus pulchellus* ticks collected on 4 days during the fall of 1999 were identified as similar to DUGV on the basis of nucleotide sequence of a fragment of the S segment genomic RNA. This virus has been designated Kupe virus.

**Figure 1:**
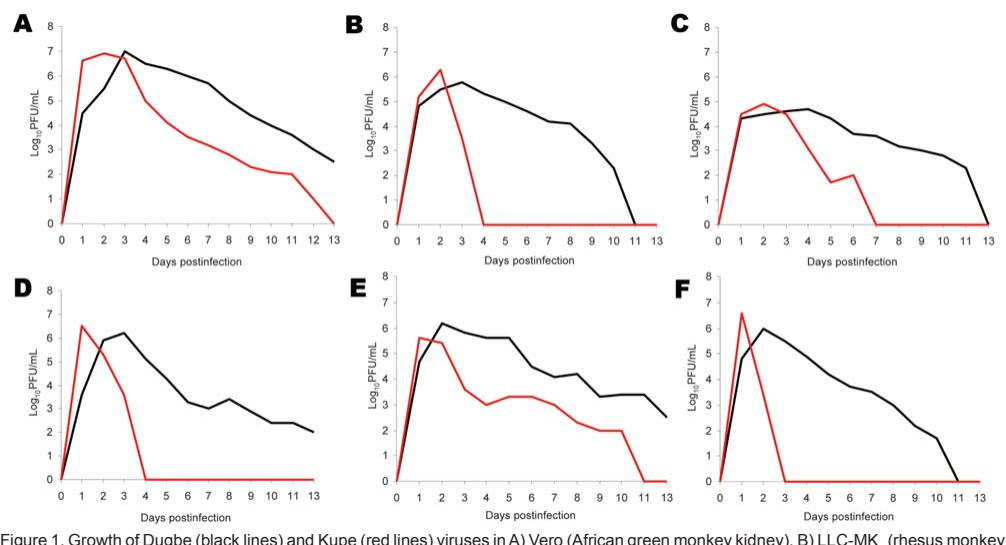
Growth of Dugbe (black lines) and Kupe (red lines) viruses in A) Vero (African green monkey kidney), B) LLC-MK_2_ (rhesus monkey kidney), C) BHK (baby hamster kidney), D) SW-13 (human adrenal cortex carcinoma), E) HeLa (human cervical adenocarcinoma), and F) HUH-7 (human hepatocarcinoma) cells in culture.

Growth kinetics of Kupe virus and DUGV were compared in 7 cell types (Figure 1). Neither virus replicated in C6/36 mosquito cells. Kupe virus and DUGV replicated in all mammalian cell types tested, and maximum titers were observed 1–2 or 2–4 days postinfection, respectively. The Kupe virus titer increased more rapidly than the DUGV titers and achieved peak titers 1–2 days earlier. The subsequent decrease in titer was also more rapid (Figure 1). In all mammalian cell types except BHK cells, we observed earlier appearance of cytopathic effects (CPE) in Kupe virus–infected cells; CPE progressed more rapidly in DUGV-infected BHK cells. However, in all but LLC-MK_2_ cells, Kupe virus caused greater overall destruction of the cell monolayer by the end of the growth curve experiment. In Vero cell plaque assays, DUGV plaques were slower to form than those caused by Kupe virus, although plaque morphology of the 2 viruses was similar (2–4 mm in diameter).

### Genome Analysis

The 3 genomic RNA segments of Kupe virus, isolate K611, were completely sequenced, ORFs were identified, and deduced amino acid sequences were determined. Similar to other viruses in this family, the ends of each RNA segment contain a conserved sequence, the terminal 9 nt of which are identical to those found in all segments of DUGV, CCHFV, and HAZV and in the S segment of NSDV (sequence of other NSDV segments not available). The S segment of Kupe virus has 1,694 nt, an ORF of 483 aa, and 5′ and 3′ noncoding regions (NCRs) of 49 nt and 193 nt, respectively. The DUGV S segment has 1,716 nt, 5′ and 3′ NCRs of 51 nt and 213 nt, and an ORF of 483 aa (*8*,*9*).

The Kupe virus M segment RNA has 4,818 nt and contains 1 ORF flanked by 5′ and 3′ NCRs of 47 nt and 121 nt, respectively. The DUGV M segment has 4,888 nt and its 5′ and 3′ NCRs are 47 nt and 185 nt, respectively (*9*). As observed in other nairoviruses, the Kupe virus M ORF, which has 1,549 aa, is longer than those of other members of *Bunyaviridae* (*9**,**10*). The Kupe virus M ORF contains 8 potential sites for N-linked glycosylation (N-gly); the DUGV M ORF contains 10 potential sites (Table 2). Kupe virus contains a unique potential N-gly site in the Gn and Gc glycoprotein regions (aa 612 and aa 1514) and was missing potential sites found at aa 30, 80, 848, and 1258 in DUGV. Further analysis is necessary to determine which of the potential N-gly sites are used in DUGV and Kupe virus proteins. DUGV and Kupe virus M segment ORFs contain a highly variable, mucin-like region near the amino terminus, as described for the genome of CCHFV (*9*,*11*). This ≈100-aa region in DUGV and Kupe virus is shorter than the 243–248-aa region identified in CCHFV, but this region in both viruses contains similarly high amino acid sequence variability, increased frequency of serine, threonine, and proline residues, and more highly predicted O-linked glycosylation than for the rest of the ORF.

**Table 2:** Potential N-linked glycosylation sites in the medium segment of Dugbe and Kupe viruses

Amino acid location*		Region†
Dugbe virus	Kupe virus	
25	16	Mucin-like, variable
30	–	Mucin-like, variable
80	–	Mucin-like, variable
142	140	Upstream of Gn
413	414	Gn
–	612	Gn
827	829	Unknown
848	–	Gc precursor
1201	1203	Gc
1258	–	Gc
1420	1421	Gc
–-	1514	Gc

*Amino acid location in the translated open reading frame. †Gn and Gc are glycoproteins.

Previous studies of CCHFV and DUGV suggest that precursors of Gn and Gc glycoproteins are produced and then post-translationally cleaved to form mature glycoproteins. Potential tetrapeptide cleavage sites for SKI-1/S1P protease or a related protease have been identified immediately upstream of the N-termini of the CCHFV (RRLL^519^– Gn, RKPL^1040^– Gc) and DUGV (RKLL^374^– Gn, RKLL^896^– Gc[predicted]) glycoproteins; similar peptides are found in the Kupe virus ORF (RRIL^375^ and RRLL^898^) (*11*–*13*). Additionally, a furin-like cleavage recognition motif (RSKR^247^) has been identified in CCHFV upstream of the amino terminus of Gn that has been shown to produce an additional glycoprotein; however, DUGV and Kupe virus do not share this motif (*14*). They contain an additional SKI-1/S1P-like cleavage motif in this region (DUGV–RRII^204^; Kupe virus–RRIL^202^).

As reported for DUGV and CCHFV, the length of the L segment RNA (12,330 nt) and ORF (4,050 aa) of Kupe virus is almost twice that of other bunyaviruses (*15*,*16*). The L RNA contains a 5′ NCR of 40 nt and a 3′ NCR of 137 nt; the 5′ and 3′ NCRs of DUGV are 40 and 104 nt, respectively. The Kupe virus ORF aa sequence shows a high degree of homology to that of DUGV, with the exception of a highly variable region (Kupe virus aa 755–896) that shows low homology (24.8%) and in which the DUGV sequence is 14 aa shorter than Kupe virus (42 nt deletion in DUGV relative to Kupe virus). In this same region, a 92-nt deletion has been shown in CCHFV relative to DUGV, and a similar deletion is observed in HAZV (*17*). All conserved motifs in the RNA-dependent RNA polymerase (RDRP) module (region 3), as well as other conserved domains upstream and downstream of the polymerase module (regions 1, 2, and 4), were conserved in the Kupe virus ORF, as shown in DUGV and CCHFV (*16*). Kupe virus L segment ORF also contains several protein motifs previously identified in DUGV and CCHFV, including an ovarian tumor–like cysteine protease domain, a DNA topoisomerase–like domain (aa 76–94), and a C2H2-type zinc finger motif (aa 608–631) (*17*,*18*). However, Kupe virus ORF did not contain the leucine zipper motif identified in CCHFV and DUGV.

### Phylogenetic Analysis

**Figure 2:**
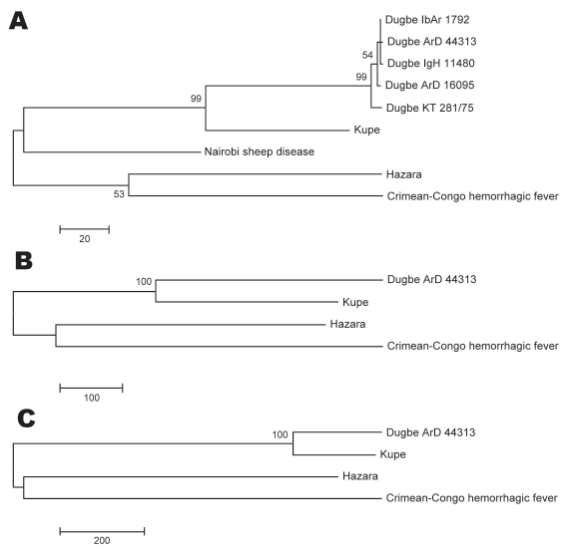
Phylogenetic trees produced by using maximum-parsimony analysis with 500 bootstrap replicates on alignments of full-length amino acid sequences of the A) small segment, B) medium segment, and C) large segment of Kupe virus with other available full-length nairovirus sequences. Scale bars indicate branch length and bootstrap values >50% are shown above branches.

Nucleotide and deduced amino acid sequences of Kupe virus segments were compared with sequences from other nairoviruses available in GenBank and with partial sequences of DUGV isolates obtained in the 1999 Kenya survey in which Kupe virus was isolated (Tables 3–6) (*3*). Comparison of full-length S segment sequences showed 68.8%–69.4% nt and 74.9%–75.5% aa sequence identity between Kupe virus and 5 strains of DUGV. Identities among the 5 DUGV strain sequences were nt 90.9%–99.4% and aa 98.1%–99.8%. Pairwise, full-length S segment nucleotide and amino acid identities among DUGV, CCHFV, NSDV, and HAZV ranged from 59.0%–64.1% and 55.3%–63.2%, respectively (see Table 3 for specific pairwise identities). A 428-nt fragment of the S segment, corresponding to Kupe S nt 44–471, was also sequenced from 26 DUGV isolates obtained during the 1999 abattoir survey (GenBank accession nos. FJ422213–FJ422238) and compared with available DUGV sequences from GenBank (Table 1) and Kupe virus. Results of these comparisons are shown in Table 6. Nucleotide and amino acid sequence identities among 5 Kupe virus isolates for a 504-nt fragment (nt 413–916) of the S segment were 95.0%–98.4% and 98.8%–100.0%, respectively (GenBank accession nos. EU257626, EU816906–EU816909). Results of phylogenetic analysis of the full-length S segment amino acid sequence alignment is shown in Figure 2, panel A. Kupe virus is shown as most closely related to DUGV, although it is distinct from the clade containing the 5 DUGV strains.

**Table 3:** Pairwise comparison of full-length nucleotide and amino acid sequences of the small segment of Kupe virus with other nairoviruses*

Virus	Kupe	Dugbe ArD44313	Dugbe ArD16095	Dugbe KT281/75	Dugbe IbH11480	Dugbe IbAr1792	NSDV	HAZV	CCHFV
Kupe		69.3	69.4	69.4	68.8	69.1	65.1	60.4	61.2
Dugbe ArD44313	75.2		99.3	91.1	98.9	99.1	63.6	60.0	59.6
Dugbe ArD16095	75.2	99.4		91.7	99.2	99.2	64.1	59.9	59.6
Dugbe KT281/75	74.9	98.1	98.3		91.0	90.9	63.3	59.1	59.0
Dugbe IbH11480	74.9	99.6	99.4	98.6		99.4	63.7	59.6	60.3
Dugbe IbAr1792	75.5	99.8	99.6	98.3	99.8		63.8	59.6	60.1
NSDV	64.0	59.9	60.1	59.5	59.9	60.1		63.5	63.1
HAZV	57.6	55.7	55.7	55.3	55.7	55.9	63.2		60.4
CCHFV	57.5	56.4	56.2	56.4	56.0	56.2	62.7	59.5	

*Nucleotide identity (%) is shown above the diagonal, and amino acid identity (%) is shown below the diagonal. NSDV, Nairobi sheep disease virus; HAZV, Hazara virus; CCHFV, Crimean-Congo hemorrhagic fever virus.

**Table 6:** Nucleotide and amino acid sequence comparisons between fragments of Kupe and Dugbe viruses*

Segment and virus	Virus		
Small (428 nt)	Kupe	Dugbe, Kenya, 1999	Other Dugbe†
Kupe		68.8–70.9	69.2–70.9
Dugbe, 1999, Kenya	67.6–69.7		89.3–97.9
Other Dugbe†	69.0	95.8–100.0	
Medium (308 nt)	Kupe	Dugbe, Kenya, 1999	Dugbe ArD44313
Kupe		63.9–65.2	65.8
Dugbe, 1999, Kenya	61.8–64.7		86.8–92.3
Dugbe ArD44313	63.7	93.1–98.0	
Large (603 nt)	Kupe	Dugbe, Kenya, 1999	Dugbe ArD44313
Kupe		81.8–82.4	81.3
Dugbe, 1999, Kenya	94.5–96.0		91.0–92.0
Dugbe ArD44313	94.5	96.0–98.0	

*Nucleotide identity (%) is shown above the diagonal, and amino acid identity (%) is shown below the diagonal. †Dugbe viruses listed in Table 1.

Full-length M segment sequences are available for only 3 of the known nairoviruses: DUGV (strain ArD 44313), HAZV, and CCHFV. Comparison of these viruses with Kupe virus M segment sequence showed 61.9%, 54.7%, and 52.1% nt identity and 57.0%, 47.7%, and 43.0% aa identity, respectively (Table 4). Additionally, a 308-nt fragment (Kupe M segment, nt 2181–2488) was sequenced from 25 DUGV isolates obtained in Kenya in 1999 (GenBank accession nos. FJ422239–FJ422263) and compared with DUGV ArD44313 and Kupe virus. Results of these comparisons are shown in Table 6. Sequence identities between 5 Kupe virus isolates for a 1,965-nt fragment of the M segment (nt 408–2372) were 90.9%–98.8% for nt and 96.0%–99.4% for aa (GenBank accession nos. EU257627, EU816902–EU816905). Phylogenetic analysis of full-length M segment amino acid sequences resulted in a tree with topology similar to that of the S segment tree (Figure 2, panel B).

**Table 4:** Pairwise comparison of full-length nucleotide and amino acid sequences of the medium segment of Kupe virus with other nairoviruses*

Virus	Kupe	Dugbe ArD44313	HAZV	CCHFV
Kupe		61.9	54.7	52.1
Dugbe ArD44313	57.0		53.7	52.5
HAZV	47.7	44.4		50.8
CCHFV	43.0	38.3	41.4	

*Nucleotide identity (%) is shown above the diagonal, and amino acid identity (%) is shown below the diagonal. HAZV, Hazara virus; CCHFV, Crimean-Congo hemorrhagic fever virus.

Full-length L segment sequences are available only for DUGV (strain ArD 44313), HAZV, and CCHFV. Comparison of these sequences with Kupe virus sequence showed 77.4%, 62.8%, and 61.5% nt identity and 89.0%, 66.3%, and 63.7% aa identity, respectively (Table 5). As expected from this data, phylogenetic analysis of full-length L segment aa sequence resulted in a tree showing Kupe virus more closely related to Dugbe virus than in the S or M segment trees (Figure 2, panel C).

**Table 5:** Pairwise comparison of full-length nucleotide and amino acid sequences of the large segment of Kupe virus with other nairoviruses*

Virus	Kupe	Dugbe ArD44313	HAZV	CCHFV
Kupe		77.4	62.8	61.5
Dugbe ArD44313	89.0		63.4	62.1
HAZV	66.3	66.1		62.3
CCHFV	63.7	63.4	64.0	

*Nucleotide identity (%) is shown above the diagonal, and amino acid identity (%) is shown below the diagonal. HAZV, Hazara virus; CCHFV, Crimean-Congo hemorrhagic fever virus.

**Figure 3:**
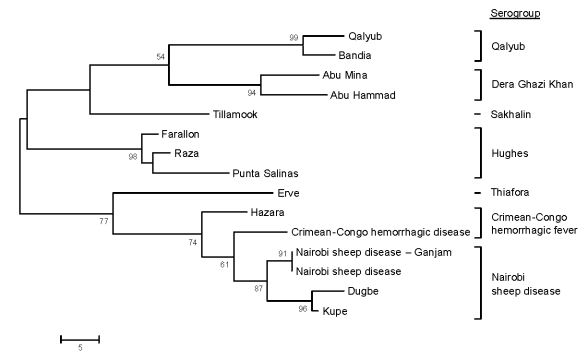
Phylogenetic tree produced by using maximum parsimony analysis with 500 bootstrap replicates on amino acid alignment of nairovirus large segment fragment (147 aa sequence translated from 441 nt sequence). Scale bar indicates branch length and bootstrap values >50% are shown above branches.

Nucleotide and amino acid sequence comparisons of a 441-nt fragment of the highly conserved L segment RDRP catalytic core domain (Kupe virus nt 6986–7426) were also made between Kupe virus and sequences of 14 other viruses representing 7 groups of the *Nairovirus* genus published by Honig et al. (*19*). A phylogenetic tree derived from the amino acid alignment of these sequences shows Kupe virus most closely related to DUGV (82.8% nt identity/95.9% aa identity), NSDV (74.9%/92.5%), CCHFV (71.9%/88.4%), and HAZV (71.7%/87.8%) (Figure 3). An additional 603-nt L fragment alignment overlapping the RDRP core domain (Kupe virus nt 7292–7894) included sequences from 26 DUGV isolates obtained in Kenya in 1999 (GenBank accession nos. EU359010–EU359035), DUGV ArD 44313, and Kupe virus. Results of these comparisons are shown in Table 6. Sequence identities among 5 Kupe virus isolates for this fragment were nt 91.2%–100.0% and aa 98.5%–100.0% (GenBank accession nos. EU257628, EU816898–EU816901).

## Discussion

Although little genetic information is available for most viruses in the genus *Nairovirus*, current classification of the diverse group of viruses in the genus is in relative agreement with available genetic analyses (*19*,*20*). Genetic information is useful in identifying emerging viruses and in analysis of relationships between viruses, especially given the segmented nature of the nairovirus genome, which can lead to generation of new viruses by segment reassortment (*21*). Within the genus, however, limited species and strain comparisons are available, making the definition of a genetic classification criteria difficult, and the segmented nature of the genome confounds the analysis. These findings are shown by a recent in-depth genetic analysis of CCHFV strains that demonstrated a high degree of genomic plasticity and RNA segment reassortment among virus strains studied (*22*).

Detailed study of the complete genome of 13 geographically and temporally diverse strains of CCHFV demonstrated nt/aa sequence identities of 80%/92%, 69%/73%, and 78%/90% for the S, M and L segments, respectively (*22*). Similarly, comparison of published full-length S segment sequences from 5 strains of DUGV isolated in Senegal, Nigeria, and Kenya between 1964 (IbAr1792) and 1985 (ArD443143) demonstrated sequence identities >90% at the nucleotide and amino acid levels (Table 3). Likewise, >89% identities were observed when a fragment of S segment sequence from these 5 strains was compared with 26 DUGV isolates from the 1999 Kenya abattoir survey (Table 3). S segment sequence identity between Kupe virus and DUGV falls well below identities observed among strains of either DUGV (Tables 3, 6) or CCHFV and is closer to that observed in S segment sequence comparisons among DUGV, CCHFV, NSDV, and HAZV (Table 3) (*22*).

Although comparison of full-length M segment sequence among multiple DUGV strains is not possible because of lack of available sequence information, sequence identities for comparison of a fragment of the M segment of DUGV ArD44313 and the 26 isolates obtained in Kenya in 1999 were >86% for nt and >93% for aa. In contrast, identities observed between Kupe virus and the DUGV sequences were considerably lower and, similar to the S segment sequence, were closer to identities observed among DUGV, CCHFV, and HAZV. In addition, differences in the number and positions of potential N-gly sites in the M segment ORF between DUGV and Kupe virus suggest substantial differences between these viruses.

Comparison of Kupe virus L segment sequences was inconclusive in determining its relationship to DUGV. Again, because of lack of available sequence information, comparison of multiple full-length DUGV strains is not possible at this time; comparison of a fragment of the L segment between the Kenya DUGV isolates and DUGV ArD44313 showed identities >91%. The relatively high full-length L segment nt/aa sequence identities of 77.4%/89.0% observed between Kupe virus and DUGV strain ArD 44313 are similar to identity levels reported among full-length L segment comparisons of CCHFV strains. This finding suggests that the L segment of Kupe virus may have been acquired from DUGV by reassortment. However, identities observed for the highly conserved 603-nt L segment fragment between Kupe virus and DUGV ArD44313 were somewhat lower compared with identities between DUGV ArD44313 and other Kenya DUGV isolates. These lower identities, combined with differences observed in the L segment variable region (Kupe virus aa 755–896), suggest otherwise.

Little is known about the ecology of Kupe virus other than its isolation from ticks infesting cattle. DUGV has been reportedly isolated from several tick species, including *A*. *gemma* and *R*. *pulchellus*, the species from which Kupe virus was isolated (*19*,*23*,*24*). In the 1999 Kenya abattoir survey, DUGV was isolated from 4 species of ticks, *A*. *variegatum*, *A*. *gemma*, *A*. *lepidum*, and *R*. *pulchellus* (*3*). Although ≈1,000 specimens each of *A*. *variegatum* and *A*. *lepidum* were collected and tested in that study, no isolates of Kupe virus were found in those species, which suggested that vector hosts for DUGV and Kupe virus may differ (*3*). Specific vector competence studies will be needed to resolve this point. The pathogenesis, if any, of Kupe virus in mammals is unknown.

Kupe virus and DUGV were observed to replicate and cause CPE in a variety of cultured mammalian cell types. Kupe virus was observed to have a more rapid increase and subsequent decrease in viral titer, an earlier onset of visible CPE, and greater destruction of the cell monolayer in most of the mammalian cells tested. These findings show that this virus is more virulent than DUGV in the mammalian cells tested.

Taxonomic classification of viruses is an evolving discipline that in early years was based primarily on morphologic characters. More recently, better classification has been obtained by using antigenic relationships and information gained from genetic characteristics. The International Committee on Taxonomy of Viruses has defined a virus species as “a polythetic class of viruses that constitute a replicating lineage and occupy a particular ecological niche” (*25*). This definition and its use in virus classification has been the subject of much discussion in the literature, and its application to newly described viruses is often difficult because of incomplete descriptive information about the new virus and other viruses in the group to which it is related (*26*,*27*).

For Kupe virus, nucleotide and amino acid sequence variation between the S and M segments of Kupe virus and DUGV, or any other genetically characterized nairovirus, was greater than expected between strains of a single virus in the genus *Nairovirus*. We also noted differences in other genetic characteristics between Kupe virus and DUGV, including M segment N-gly sites, L segment variable region, and NCR length variations. This evidence, combined with increased virulence of Kupe virus in cultured mammalian cells and potential differences in vector hosts, shows that Kupe virus is substantially different from, although closely related to, DUGV and is a new virus in the genus *Nairovirus*. However, further studies are necessary to determine the hosts, vectors, and geographic range of Kupe virus along with its virulence as a human or animal pathogen. Such information will aid in appropriate classification of this new virus.
